# 2D inversion of electrical resistivity investigation of contaminant plume around a dumpsite near Onitsha expressway in southeastern Nigeria

**DOI:** 10.1038/s41598-021-91019-3

**Published:** 2021-06-04

**Authors:** Charles Chibueze Ugbor, Ifeanyi Emmanuel Ikwuagwu, Osim Jethro Ogboke

**Affiliations:** 1 Department of Geology, University of Nigeria, Faculty of Physical Sciences, Nsukka, 410001 Enugu State Nigeria; 2 Nigerian National Petroleum Corporation (NNPC-NAPIMS), 38 Gerard Road, Ikoyi, Lagos, Nigeria

**Keywords:** Environmental sciences, Hydrology

## Abstract

The study tries to utilize vertical electrical sounding (VES) and 2D resistivity tomography to evaluate the region of influence of the leachate plume on the groundwater around a dumpsite at Onitsha expressway, southeastern Nigeria. The borehole log data were acquired and their respective geoposition logged with Garmin GPS device. In addition, four 1D (VES) soundings and 2D electrical profile data were acquired in the field utilizing the Schlumberger and Wenner profiles respectively. Petrozenith PZ-03 Resistivity meter was used to acquire the electrical data, while RES2DINV and WinResist software were used to interpret the 2D and 1D data respectively. The resulting geoelectic layers were correlated with the borehole logs and were interpreted according to their resistivity distribution. Results of the 2D inversion at profiles 1 and 3 showed low resistivity zones indicating influence from the leachate plume. Profiles 2 and 4 gave low resistivity zone within 14.6 and 44.3 Ωm from surface to between 0.375 and 3.60 m depths indicating influence from leachate plume. Likewise, profiles 1 and 3, which penetrated groundwater, also showed very low resistivity with resistivity ranging from 3.12 to 8.7 Ωm, from surface to few meters depths. This indicates that it has been polluted by the leachate. In contrast, Profiles 2 and 4, from the 2D inversion, has no leachate influence on the groundwater. The VES result showed that the depth to the water table at location 1, 2, 3 and 4 are 21.7 m, 17.9 m, 15.9 m and 12.2 m respectively, with the leachate plume flowing in the southeast direction in line with the groundwater flow direction.

## Introduction

Solid waste disposal sites frequently create environmental challenges, which also adversely affect the settlers within and around the vicinity of the area. This includes the degradation of the soil and pollution of the surface and groundwater. Leachate from wastes usually constitutes all the materials that exudes from household or industrial wastes sites, which contain high concentration of the dissolved/suspended materials. These materials when washed out/leached from the wastes go into solution in water which is a universal solvent. The region of influence or extent to which this leachate had progressed from the source over time is being considered here as the leachate plume. The composition of a landfill is dependent on the type, quantity, and composition of the solid waste disposed. These may be from industrial, household, or a combination of both. The leachate is also affected by the type of disposal system, the edaphic, hydrological, and biochemical interactions within the materials, and the environment. The result of this interaction could pose a danger to the inhabitants if it results in pollution of the groundwater. This is because the inhabitants depend on the available water for both household and industrial use, likewise, the aquatic and plant life in the area. A good number of health disorders such as anemia, heavy metal poisoning, behavioral and genetic disorder among others could result from infiltration of polluted water from improperly disposed waste^1,2^. Poorly planned waste management system in a rapidly industrializing city like Obosi–Onitsha can potentially aggravate the challenge of containing the influx of industrial and household waste in the area. The high precipitation during the 5–6 months of the rainy season in the area supplies the solvent that dissolves and conducts the leachate through the subsurface and to potentially contaminate or pollute the groundwater^3,4^. Obosi town and the environments being a fast developing town in Idemili Local Government Area of Anambra state, requires adequate monitoring of the environment as key to proper town planning and hydrological management strategy in the area. This will help to mitigate any deleterious effect of uncontrolled leachate infiltration and contact with the groundwater on human health.

**Figure 1:**
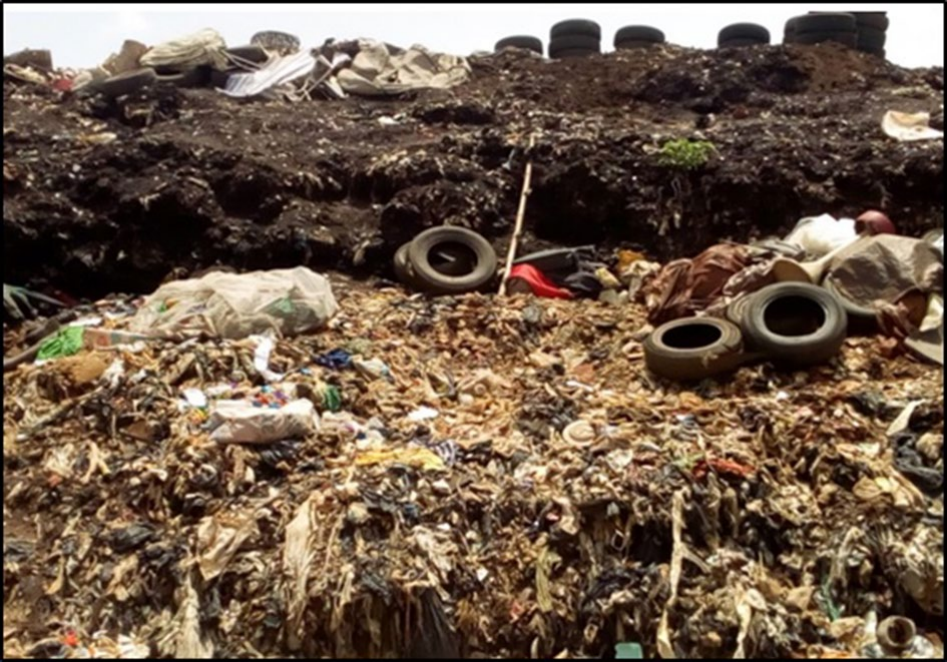
Photo showing the compositions of the refuse on the surface of the dumpsite.

Figure 1 shows the composition of solid waste deposited in the dumpsite, which contains several types of industrial, domestic and solid wastes. Examples are damaged tyres and batteries, polythene bags, plastics bottles, decayed food items, fluorescent tubes etc.

The relatively higher ionic content of leachate makes the resistivity of a typical leachate plume correspondingly lower than the surrounding areas. The electrical resistivity (ER) method has been chosen for this study over other geophysical methods because the target anomaly would more easily respond to electrical conductivity since the migrating leachate/contaminants are more conductive that the surrounding area^5^. Hence resulting electrical resistivity anomaly can easily be mapped using simple electrical resistivity equipment. Geophysical methods such as seismic, gravity and radioactive methods do not suit this application because they do not respond to anomaly resulting from varying concentration of the contaminants. The electrical resistivity method is applied in delineating areas affected by the leached substances. This is because of a high ionic concentration and conductivity of the leachate which is easily measured using the electrical method. Several authors have studied dumpsites using electrical resistivity and sometimes with a combination of other methods. The Aurum solid waste landfill site near Edmonton, Alberta was studied by a research team using electrical resistivity method^6^. They reported that the regions affected by the leachate from the landfill recorded relatively higher electrical conductivity than the areas farther from the landfill that had not been affected by the leachate. Enikanselu^7^ studied the dumpsite-induced groundwater contamination around Giwa-Okearo, Ogun State, Nigeria using electrical resistivity method. He found that the groundwater in areas closest to the dumpsite, and up to 72 m away, are characterized by low resistivity values indicating influence of the contaminant. He also reported that a time lapse monitoring of the leachate plume shows that it spreads by 652 m^2^ per year. Ugwu and Nwosu^8^ studied the effect of waste dumps on groundwater in Choba area, Port Harcourt, Nigeria, using electrical sounding method and chemical analysis. The results from the two locations studied showed that the area close to the dumpsite, and with higher electrical conductivity, is contaminated while the areas outside the dumpsite had relatively lower electrical conductivity and hence uncontaminated by the leachate. They also stated that the result is in line with the chemical analysis of the samples taken from the area, where the contaminated areas had higher concentration of the chemical elements. In their separate investigation, Omolayo and Tope^9^ examined the generation, migration and impact of the leachate plume on groundwater and the soil at Ibadan, southwestern Nigeria using 2D electrical imaging and element analysis, They concluded that the values of both the total dissolved solids and the nitrate were above the WHO standard, hence pose health risk to the inhabitants. Salami et al.^10^ studied the impact of a closed dumpsite on the environment at Oke Afa, Lagos state, Nigeria. They stated that the dumpsite did not significantly affect the water resources in the area since the parameters studied fell within the recommended concentration by the World Health Organization (WHO) and the leading environment agency in Nigeria, the National Agency for Food and Drug Administration and Control. Wijesekara et al.^11^ studied the subsurface of an open dumpsite at Kandy, Sri Lanka using direct current resistivity method to identify possible subsurface flow path and direction of the leachate. They reported that the flow path of the leachate is confined along the low resistivity zone close to the surface with no separate plume at the downstream.

Ganiyu et al.^12^ had applied electrical resistivity imaging to map the leachate plume migration at Lapite dumpsite in Ibadan, southwestern Nigeria. They had reported that the leachate plume had high conductive values than the surrounding areas unaffected by the leachate. Emujakporue^13^ investigated the contaminants in a dumpsite at the University of Port Harcourt, Nigeria, using self potential (sp) method of geophysical investigation. He reported that the relatively lower electrical potential, at the area affected by the leachate, than at the neutral area, is attributed to the differences in the electrochemical and electrokinetic processes in the area. According to him, these resulted in the elevated values of the electric potential in the leachate-influenced areas. Maurya et al.^14^ applied 2D and 3D resistivity study of a leachate as potential groundwater contaminant. They demonstrated that the methods were useful in delineating the contaminants in the area. Ifemeje et al.^15^ studied the contaminant profile of the dumpsite based on the physicochemical and element composition. They reported that there were significant increase in the physicochemical properties at the active part of the dumpsite than the abandoned part of the dumpsite. They concluded that the leachate contributed significantly to the contamination of the groundwater in the area. Osele et al.^16^ studied the aquifer characteristics and groundwater potential of Onitsha and the environs using vertical electrical sounding (VES) technique. Using a sketch diagram, they characterized the subsurface by correlating the two datasets from the lithology derived from the borehole data and the geoelectric sections from the VES sounding interpretation. They showed that the two datasets are correlatable and were able to discriminate between vertical discontinuities based on lithology and electrical conductivities of the subsurface. They effectively determined the aquifer characteristics and the groundwater potential of the area as very promising hydrogeological system.

In addition^17^ carried out an integrated physicochemical and hydrogeochemical assessment of the study area employing well data. They reported the presence of heavy metals in concentration in excess of the WHO standards. They also showed that there was significant correlation between the high electrical conductivity and heavy metal concentration. They concluded that the area was affected by the leachate from the dumpsite that potentially affected the groundwater. Their method does not show the extent of the pollution in the area or the leachate plume in the identified main dumpsite. Therefore no attempt was made to characterize the entire study area based on the more versatile 2D tomographic imaging with wider areal coverage than the limited borehole points data. Nontheless, Hamzah et al.^18^ had used 2D and 3D electrical resistivity and chemical analysis to study the leachate migration at Sungai Sedu landfill underlain by the Holocene Gula and Berua Formation. They had attributed the low resistivity to the leachate plume from the landfill.

The present study aims at utilizing electrical resistivity method to map zones of possible contamination by a dumpsite located along the Onitsha Expressway, southeastern Nigeria. The study is carried out to ascertain the extent of the leachate over time from the time the contaminant started leaving the source to the surrounding till present. The contaminant plume being assessed is therefore the extent, or zone of influence, of the leachate over time from the initial time till present. The objectives of this research include determining: the depth to water table, delineating the contaminant leachate (zone of influence of the leachate over the years) using 2-D electrical resistivity tomographic sections and establishing the flow direction of leachate to guide future groundwater practice in the area.

## Location, topography and climate of the study area

**Figure 2:**
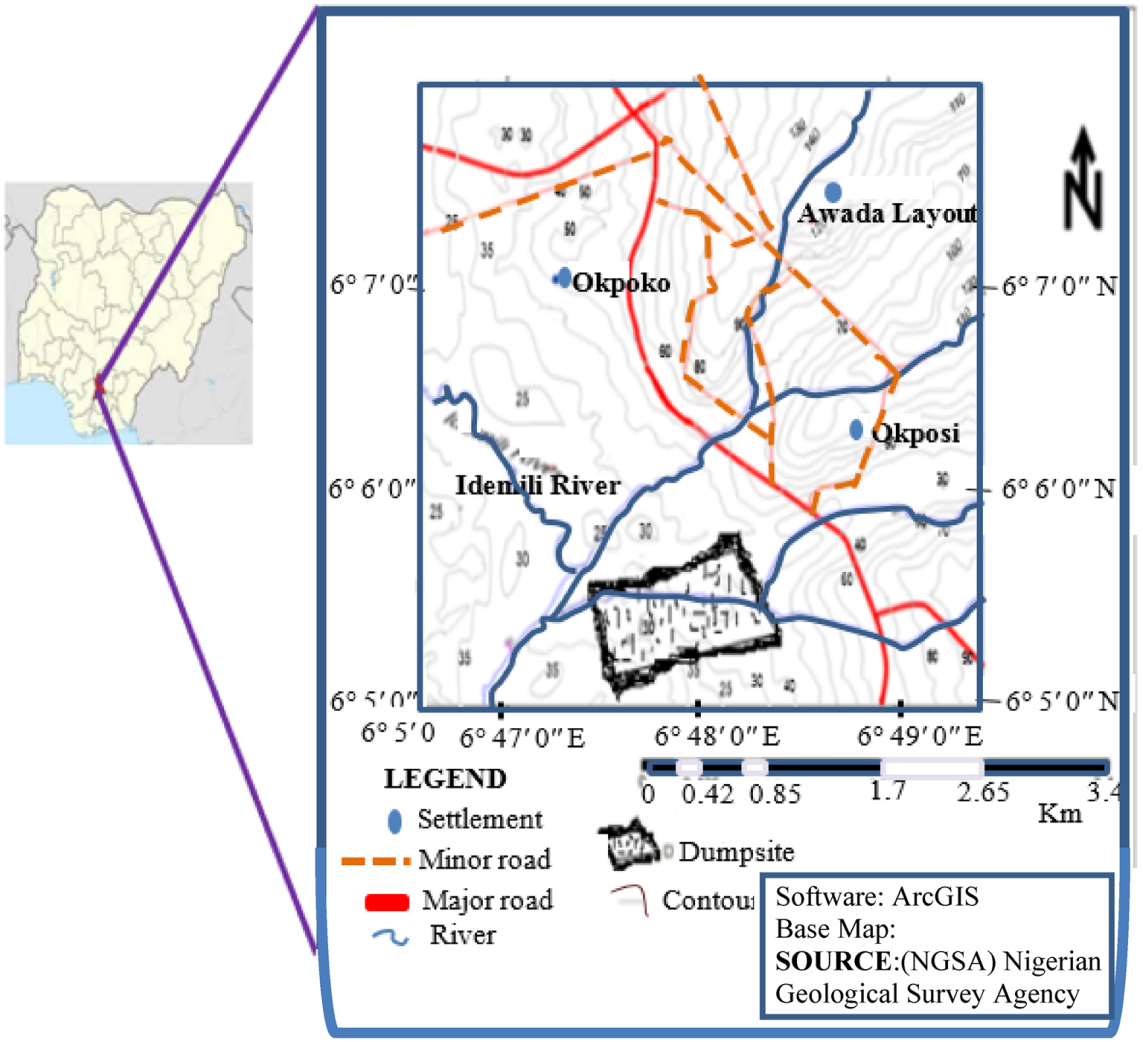
Location map of the study area produced from the field data and the topographic map showing the location of the dumpsite and the accessibility, drainage, and topography. (ArcGIS info: https://esri.uconn.edu/software/arcgis-student/download/. https://link.springer.com/referenceworkentry/10.1007%2F978-0-387-35973-1_68).

The study area is located opposite the National Metallurgical Institute (NMI), along the Onitsha Expressway. It is within the Idemili North Local Government Area of Anambra state, southeastern Nigeria. It is bounded by latitudes 6° 5′ 0″ and 6º 8′ 0″ N and longitudes 6° 46′ 30″ E and 6° 49′ 30″ E (Fig. 2). Apart from the residential areas, several industrial activities and outlets surround the dumpsite, measuring about 1.7 km × 0.65 km. These include petrol/gas dispensing stations, motor spare parts shops, mechanical, and electrical workshops and health centers. Human and industrial activities in these areas generate large volume of waste in the area and they find themselves in the open dumpsite under study.

The area has an undulating topography ranging from gently sloping low lands to numerous slightly steep highlands (Fig. 2). The topography and geomorphology of the place reflect the tectonic and geological events that occurred over a long period. This had continued to shape the geomorphic landscapes. The area is drained by the Idemili River, which flows westwards into the major lower River Niger drainage basin. Tributaries of Idemili River rise from the highlands and flow swiftly into the Idemili River actively caving deep gullies.

The study area is situated within the sub-equatorial south climatic region. It is characterized by uniformly high temperatures, seasonal distribution of precipitation, and high relative humidity. This includes dry, and dusty harmattan which occurs mostly between the months of November and March. The wet season occurs between the month of April and October, with a brief intervening dryness during some periods in the month of August. The average annual rainfall is 1500 mm. The highest temperature is between February and March, and the lowest is between 18.2 and 23 °C in August and September^19^. According to Igwe and Chukwura^17^, the elevated temperature, which varies from 27.2 and 35 °C, is prevalent with both northeastern and southwestern winds. The southwestern trade winds is laden with moisture from the South Atlantic Ocean. On the other hand, a dry, hot, and dirty trade wind emanates from the Sahara deserts. The coupled circulation patterns among these twin trade winds thus control the weather in the area.

## Geology and hydrogeology of the study area

**Figure 3:**
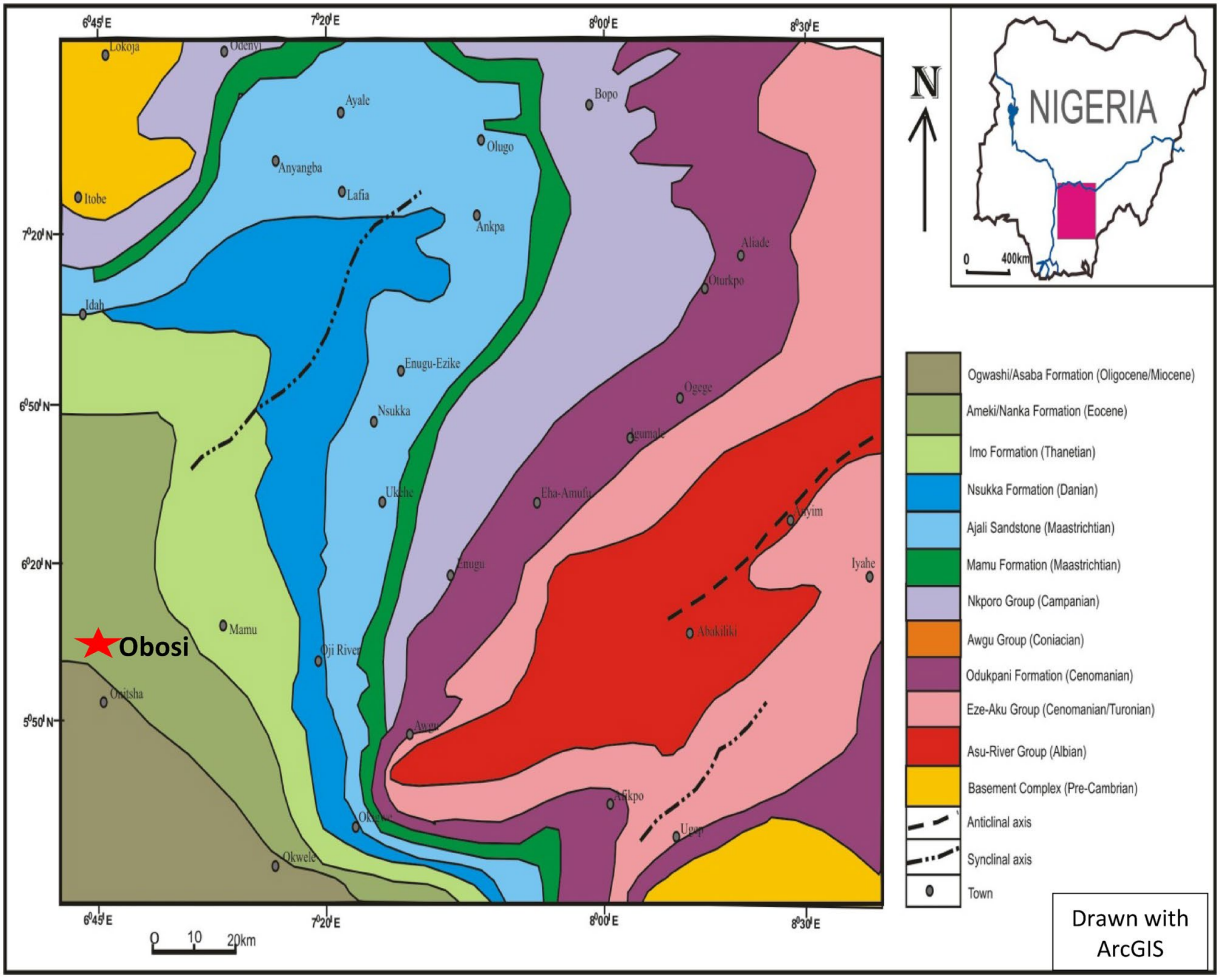
Geologic map of southeastern Nigeria showing the study area marked in red star (modified from^20^). The study area is seen here (Obosi) is situated within the Ameki Formation. Source: https://www.researchgate.net/publication/320191324/figure/download/fig1/AS:545562816909312@1507083719359/Geologic-map-of-southeastern-Nigeria-showing-the-study-area-modified-from-Hoque-1977.png.

The study area lies within the pro-delta sediments of the Niger Delta basin that was formed during the Late Tertiary depositional cycle. Obosi and the environs are underlain by the Middle Eocene sediment of tertiary period called the Ameki Formation. The sediments were deposited in a fluvio-deltaic environment. It constitutes the main bulk of the Eocene strata overlaying the Imo Shale which was deposited during the Late Palaeocene. Figure 3 shows the location of the Obosi near Onitsha, in the southeastern Nigeria sedimentary basins. Ameki Formation consists essentially of micaceous siltstone, fine-grained sandstone, and medium-grained sandstone. In some horizons, the sediment consists of a series of highly fossiliferous greyish green sandy clay with calcareous concretions and white clayey sandstone. The lithological groups have been identified in parts as consisting of fine to coarse grains in the lower parts and fine to coarse grains cross bedded sandstone, and sandy clays in the upper parts.

The stratigraphic succession of the southern Nigeria sedimentary basin shows the Imo/Ameki Formation are the first set of a pro-delta sediment sequence within the inland basin of the Niger Delta basin (Fig. 3), The Ameki Formation, being one of the inland basins, is stratigraphically a time equivalent of the Agbada Formation of the Niger Delta basin. The Imo Shale/Ameki Formation is uncomformably overlain by the Ogwashi–Asaba Formation. The contact between these two formations can be discerned by the occurrence of thin layers of lignite^20^.

The Ameki Formation is a regressive cycle deposit. Hydrologically, the Ameki Formation provides a prolific unconfined aquifer system in the area. Shallow wells and boreholes, with depth to water table range of 40–50 m, dominate the area. It has high yield and become much shallow during the rainy seasons^21^. The interpreted lithologic layer from the vertical electrical soundings confirms the presence of thick sequence of sandstone which is the target of the current investigation. The formation is capped by a lateritic layer due to weathering. It is underlain by sandy layers with some intercalation of gravels and thin clay^22^.

**Figure 4:**
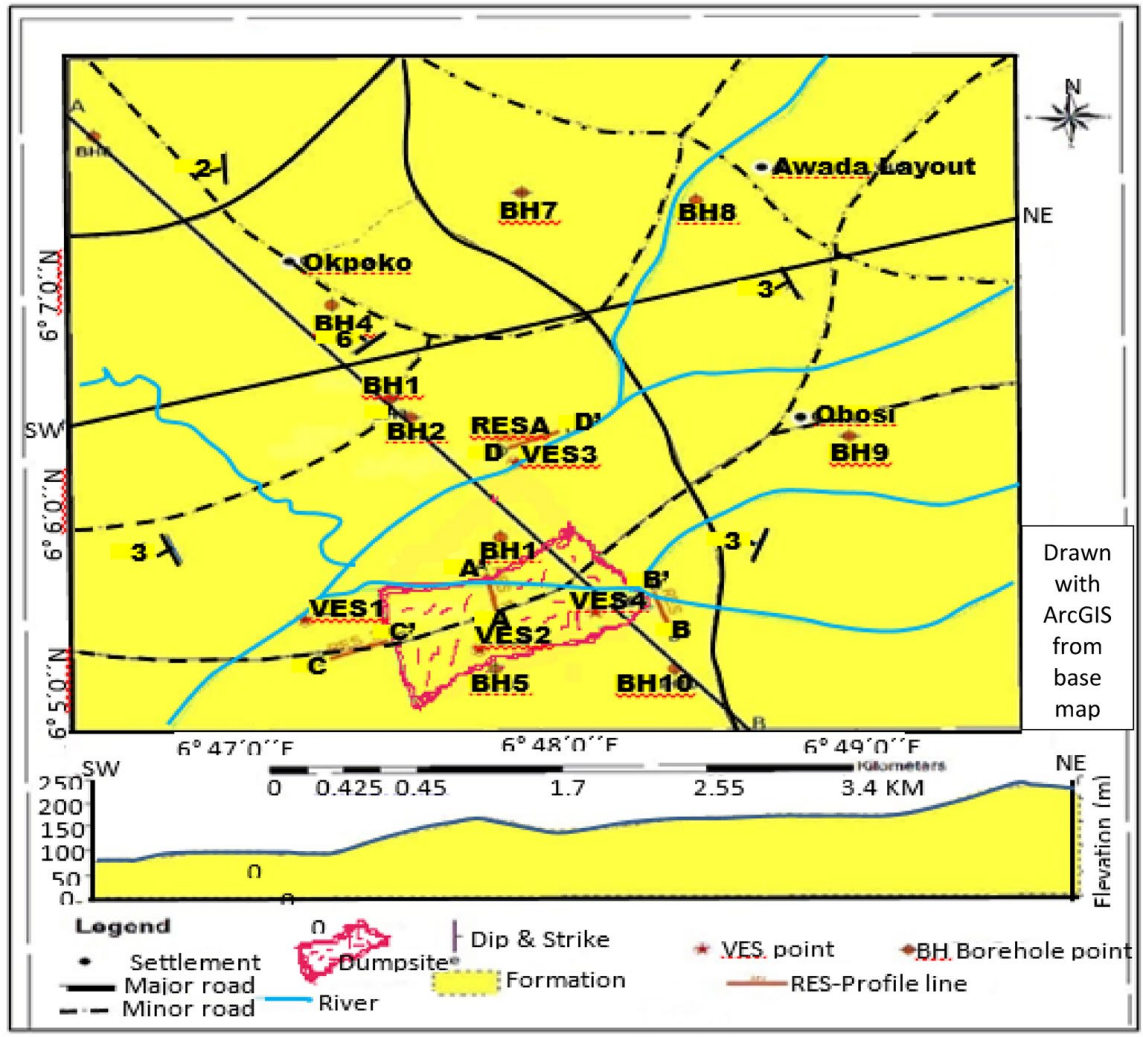
Geologic map of the study area showing the accessibility, drainage, topography and sampling points for VES, borehole and dumpsite, drawn from base map sourced from NGSA with ArcGIS software.

Figure 4 shows the Geologic map of the study area (Obosi and the environs) with the drainage pattern and location of the sampling points. The sampling points for each data acquisition were designed to cover the areas within and outside the dumpsite to help in the evaluation of the extent of the influence of the leachate. The data include the VES, Resistivity (RES) profile, and Boreholes (BH)) data. The sediments strike, mostly, in the North–South to Northwest–Southeast and dips generally towards the West or Southwest direction. The area is characterized by gently dips ranging from 3° to 6°.

## Materials and methods

### General survey

The materials used for the study include field equipment such as Compass, Garmin Global Positioning System (GPS), Electrical resistivity meter, software including Res2Dinv and WinResist. This study followed a field data acquisition and data analysis approach. The detailed fieldwork was preceded by desk study and reconnaissance survey. Available literatures on the study area were studied to get the pre-knowledge of the geology of the area. Possible access roads and outcrops were noted and field design for the geophysical survey was decided after the initial field visit.

### Geophysical survey

Two electrical configuration arrays were deployed for this study. The Wenner and Schlumberger arrays were chosen to determine the lateral and vertical variation of the leachates respectively. The Wenner array was designed to monitor the lateral variation of the leachate/contaminants through monitoring of the electrical conductivity anomaly^15^ The Schlumberger array was chosen to sample the variation of electrical properties of the subsurface, hence the area of influence of the leachate vertically/at depth^23^. The two sets of data provided an areal geoelectrical model of the subsurface within and around the dumpsite.

#### Wenner profile (RES)

For the two dimensional imaging, the Wenner array method was conducted along four profiles. In the field, the technique was achieved by sending a direct current into the ground through a pair of current electrodes, while the voltage drop was measured via another set of potential electrode pair. For each profile, a constant electrode spacing of 1a, 2a, 3a, 4a, and 5a each for both current and potential electrode were used sequentially.

For an electrode spacing of 1a, the spacing distance of current and potential electrodes are equal and are shifted at successive readings along the spread of 100 m. The same procedure was repeated for each of the electrode spacing of 2a, 3a, 4a, and 5a, separately. Where ‘a’ is the electrode spacing, and equal to 3 m.

After acquiring the data over the survey profiles, the variation in the resistivity values is employed in the interpretation of the leachate plume through an inverse modeling following the work of^23^.

#### Vertical electrical sounding (VES)

The Schlumberger array method was used to estimate the depth to the water table employing the vertical electrical sounding technique. In the field, potential electrodes were kept relatively constant while the current electrodes were successively leapfrogged outwards about the mean position of potential electrodes. Readings were taken at each successive position of the current electrode. As the current electrode spacing increases along the profile, the current penetration increased, thereby successively sampling greater depths. This process was continued until the maximum AB/2 was reached.

The data obtained using the Schlumberger configuration were subjected to 1D resistivity interpretation using the WinResist software. The data yielded 1D geoelectric layers, showing the vertical variations in the electrical conductivity in the area, The result of the interpretation of the VES consists of a set of geolectric layers showing the different geoelectric layers, thickness, and electrical resistivity at each layer. Usually, the data as quality-checked for consistency. The data is then input into the software following the processing and interpretation procedure to arrive at the final result.

The data obtained from the Wenner configuration were subjected to 2D tomographic interpretation using the Res2Dinv software. The data were quality-checked and input into the software and following standard processing and interpretation procedure the final result was displayed. The 2D model is a kriging of the model subsurface in accordance with the variation of the values in the electrical properties of the subsurface under study.

A sketch of litho-section of the borehole information was done geospatially considering the relative positions of the borehole and VES sampling points in the study area. The lithology from the boreholes were thus cross correlated with the geoelectric layers from the VES interpretation data in line with^16^. A correspondence of the geoleectric layers and the electrical characteristics enable the interpretation of the layers in terms of the electrical characteristics across the area.

The combined results were combined to generate a geo-electric model representing the areas affected by the leachate.

## Results and discussion

### 2D electrical resistivity survey

**Figure 5:**
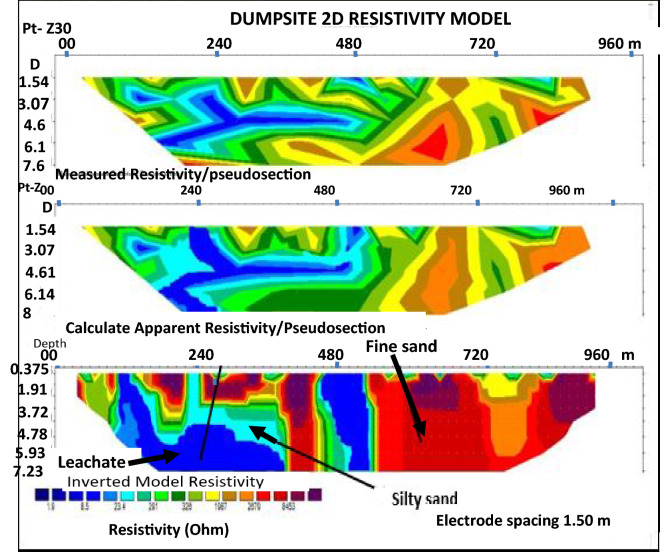
2D inversion of the Wenner-array data along profile 1 showing variation in resistivity in the subsurface, with blue colour representing least resistivity and red colour showing highest resistivity zone.

**Figure 6:**
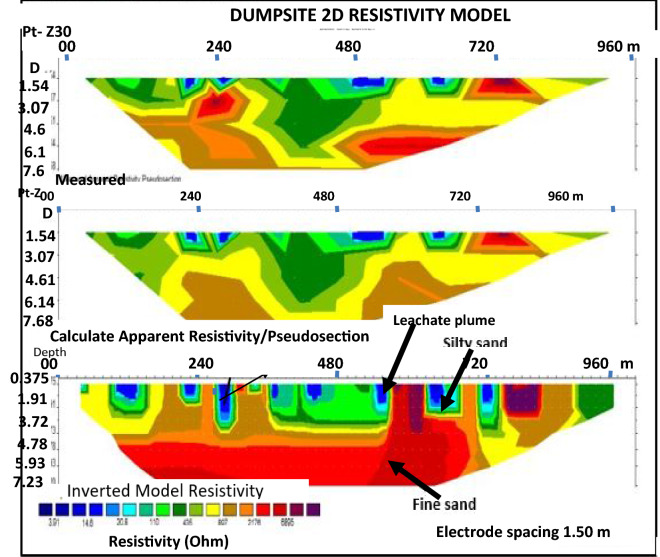
2D inversion of the Wenner-array data along profile 2 showing variation in resistivity in the subsurface, with blue colour representing least resistivity and red colour shoing highest resistivity zone.

**Figure 7:**
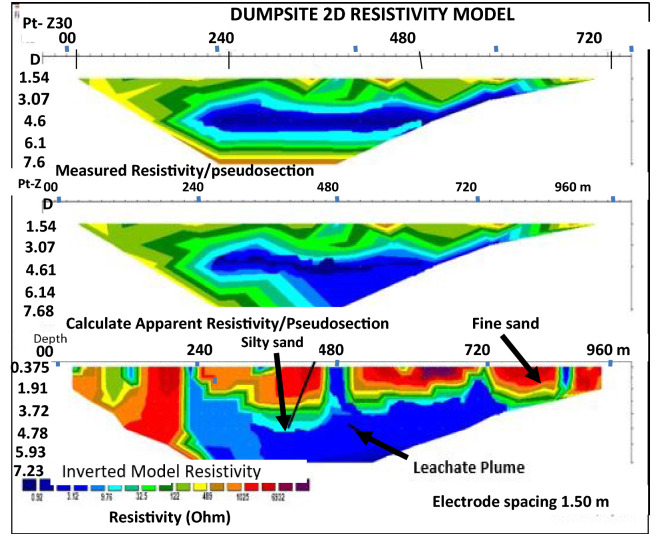
2D inversion of the Wenner-array data along Profile 3 showing variation in resistivity in the subsurface, with blue colour representing least resistivity and red colour shoing highest resistivity zone.

**Figure 8:**
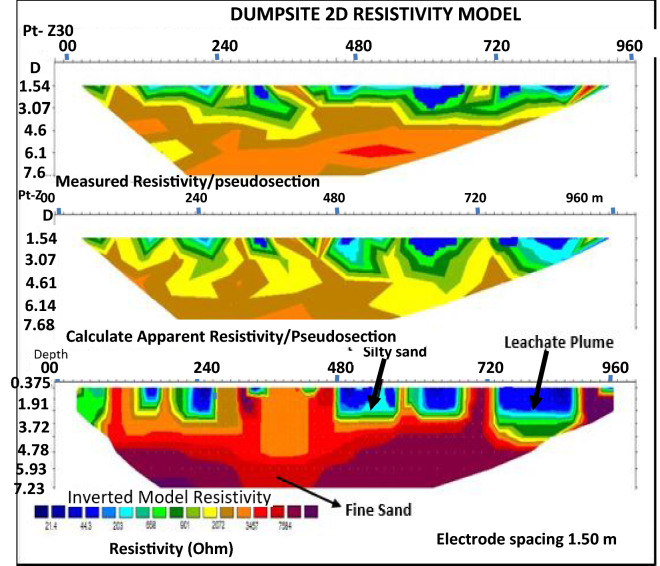
2D inversion of the Wenner-array data along profile 4 (control) showing variation in resistivity in the subsurface, with blue colour representing least resistivity and red colour showing highest resistivity zone.

Figures 5, 6, 7 and 8, show the inversion of Wenner-array data along profiles 1, 2, 3 and 4 respectively.

Profile 1. This was acquired in a NE–SW trend across the dumpsite. A resistivity inversion was obtained after 30 iteration with a 5.3% RMS error as shown as a pseudo section in Fig. 5. The apparent resistivity, measured in in ohm-metre, (Ωm) is plotted against the pseudo-depth (in m). A low resistivity zone of > 8.7 Ωm, shown in dark blue colour, occurs near the surface with depth between 0.375 to 7.20 m. This indicates the leachate plume had polluted the groundwater in this zone. This is due to the relatively low resistivity values as calibrated against the surrounding areas. A high resistivity zone of > 8453 Ωm occurs near the surface with depth ranging from 0.375 to 4.21 m. This is observed at the Northern and Western part of the section. This high resistive zone near the surface represents the uninvaded zone by the leachate. The zone, coded in red colour, with a high resistivity value of > 8453 Ωm, is essentially composed of fine sands. At depth of 0.375–7.20 m, a resistivity zone ranging in value of > 23.4 Ωm is here coded in light blue colour. This zone is interpreted as being underlain by silty sand.

Profile 2 is located at the southeastern end of the study area. It is measuring 100 m in length, and runs in the North-Western direction as shown in Fig. 6. The 2D pseudo-section from the inversion was obtained after an iteration of 30, with a 7.9% RMS error. The apparent resistivity (Ωm) is plotted against pseudo-depth (m). A low resistivity zone of > 14.6 Ωm was coded as dark blue colour. This zone is identified close to the surface at depth ranging from 0.375 to 3.1 m and indicates the zone of leachate plume invasion. In this case, the topsoil has been contaminated. A high resistivity zone with a range of > 2176 Ωm, and coded red colour, This existed near the surface with depth ranging from 0.375 to 3.7 m to the West and East of the section. This zone was interpreted as being as a result of the presence of high resistive chemical compounds. The migration near the surface is an indication that it is less dense. The lithology is inferred as fine sands. A low resistivity zone of value of > 20.8 Ωm, and coded as light blue colour, is identified close to the surface. This indicates the presence of silty sand that probably hindered easy further penetration of the leachate.

Profile 3 was acquired in a NE–SW trend direction of the dumpsite. The pseudo section (Fig. 7) shows the resistivity inversion results following 30 iteration, with 8.5% RMS error. The apparent resistivity (Ωm) is plotted against pseudo-depth (m). The Profile is located at the southwestern end of the study area, measuring 100 m in length. It runs in the southeast direction. A low resistivity zone of > 3.12 Ωm and coded in dark blue colour. It existed close to the surface with depth ranging from 0.375 to 7.2 m. This indicates that the leachate plume has penetrated beyond the topsoil into the ground. A high resistivity zone of > 1025 Ωm, and coded red colour. This occurred near the surface with depth ranging from 0.375 to 5.3 m at the eastern part of the section. This zone is interpreted as being high resistive near the surface. This is an indication that it is less dense and is interpreted as fine sands. Also at depth range from 0.375 to 5.1 m, a resistivity zone with resistivity value of 9.76 Ωm, and volour-coded light blue. This zone indicates silty sand, which promotes the leachate plume infiltration thereby enhancing its contamination of the environment.

Profile 4. It is located at the eastern end, 1 km away from the dumpsite, measuring 100 m in length, and runs in the North–South trend. The pseudo section (Fig. 8) shows the resistivity inversion results at an iteration of 30 with 29.6% RMS error. The apparent resistivity (Ωm) is plotted against the pseudo-depth (m). From the pseudo section, a low resistivity zone of > 44.3 Ωm, coded blue in colour, was isolated close to the surface with depth from 0.375 to 3.3 m. This area indicates that the leachate plume has contaminated the topsoil. A high resistivity zone of > 7584 Ωm, coded purple colour, existed near the surface. It occupied the bottom of the section with depth ranging from 0.375 to 7.20 m from South to Eastern part of the section. This zone is thus of high resistivity. It represents the presence of a denser material as the leachate displaces them as they move downwards in the dominantly clayey sandstone. The light blue coloured zone indicates silty sand, which impedes the migration of the leachate plume. The migration of the leachate plume has not affected the groundwater in this area. The red coloured zone found at the bottom indicates fine sand lithology.

### 1D vertical electrical sounding (VES)

**Figure 9:**
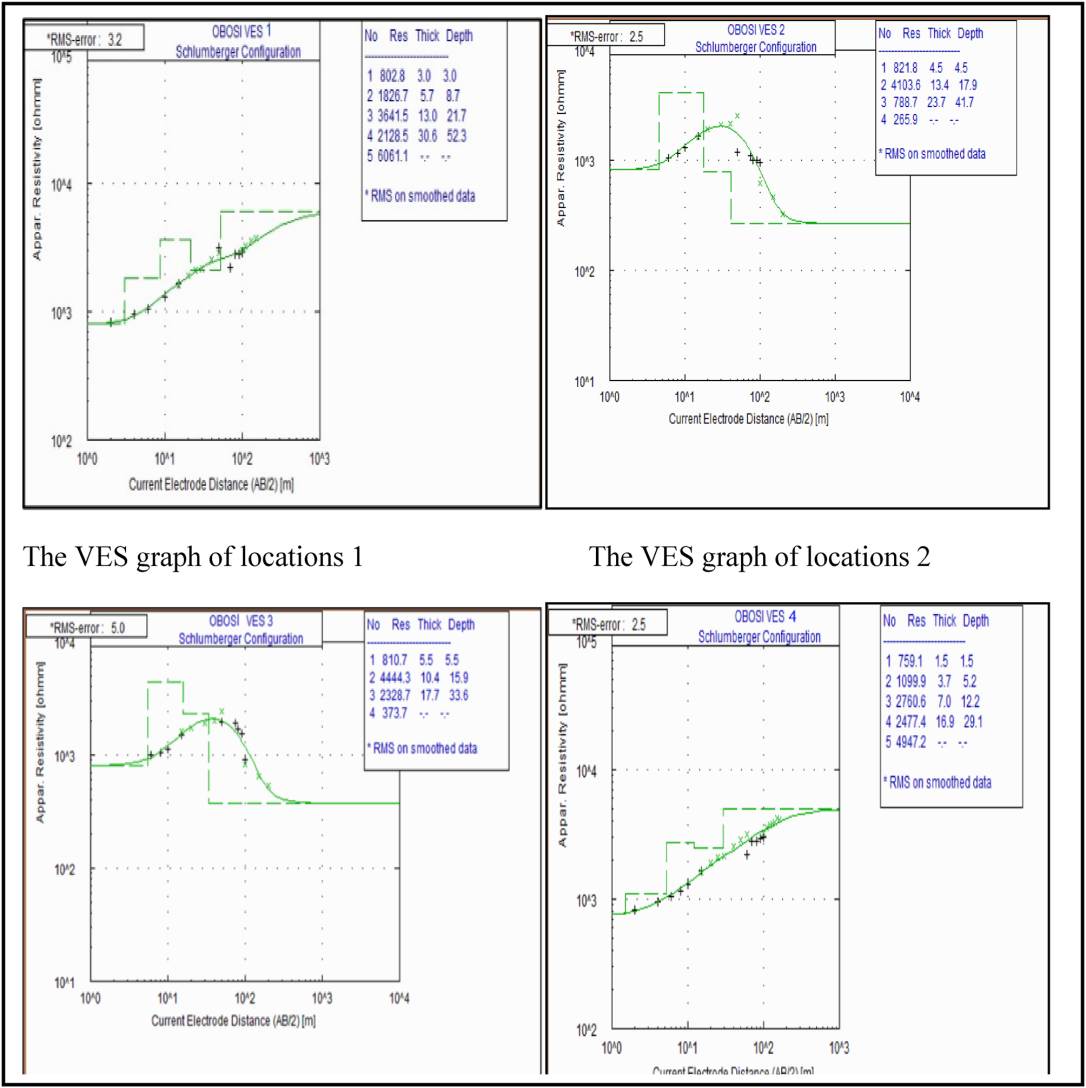
Vertical electrical sounding of locations 1, 2, 3, and 4.

Figure 9 shows the results of the four vertical electrical sounding (VES) conducted at locations VES1, VES2, VES3 and VES4. (See also Fig. 4). The curve is a plot of the apparent resistivity versus the spacing (AB/2) or distance below the subsurface. The VES curve at location VES1 depicts a type A curve of five layers. The 4th layer has a resistivity value of 2128.5 Ωm with thickness and depth of 30.6 m and 52.3 m respectively. The 5th layer has a resistivity of 6061.1 Ωm though normally, as the last layer, the entire thickness was not penetrated. This layer is interpreted to consist of coarse sands with interconnected pore spaces and has a higher resistivity value than layer 4. The 5th layer is the aquifer and shows that the depth to the water table which is 52.3 m.

VES graph at location VES2 depicts type K curve and has four layers. The 3rd layer has a resistivity value of 788.7 Ωm. The thickness and depth is 23.7 m and 41.7 m respectively. This layer contains coarse sands whose pore spaces are interconnected. The resistivity value of layer 4 is 265.9 Ωm which is lower than layer 3. This shows that the water maybe saline (NaCl rich) and highly conductive than that of layer 3. The depth to the water table is 41.7 m.

At location 3, the VES graph has a type K curve and is made up of foue layers. The 3rd layer has a resistivity value of 2328.7 Ωm. The thickness and depth is 17.7 m and 33.6 m respectively. This layer contains coarse sand with interconnected pore spaces. The resistivity value of layer 4 is 373.7 Ωm which is lower than that of the 3rd layer. This shows that the water may be saline (Nacl rich) and more highly conductive than that of layer 3. The depth to the water table is 33.6 m.

The VES graph at Location VES4 is a type A curve and is made up of five layers. The 4th layer has a resistivity of 2477.4 Ωm. The thickness and depth of 16.9 m and 29.1 m respectively. The 5th layer has a resistivity of 4947.2 Ωm though the entire depth and thickness could not be determined since it is the last geoelectric layer. Based on the correlation with the borehole log, the 5th layer contains coarse sands whose pore spaces are interconnected and has a higher resistivity value than the 4th layer. This implies that the 5th layer may contain fresh water while the water in the 4th layer may be saline. This layer shows the depth to the water table which is above 29.1 m.

### Correlation of VES and borehole data

**Figure 10:**
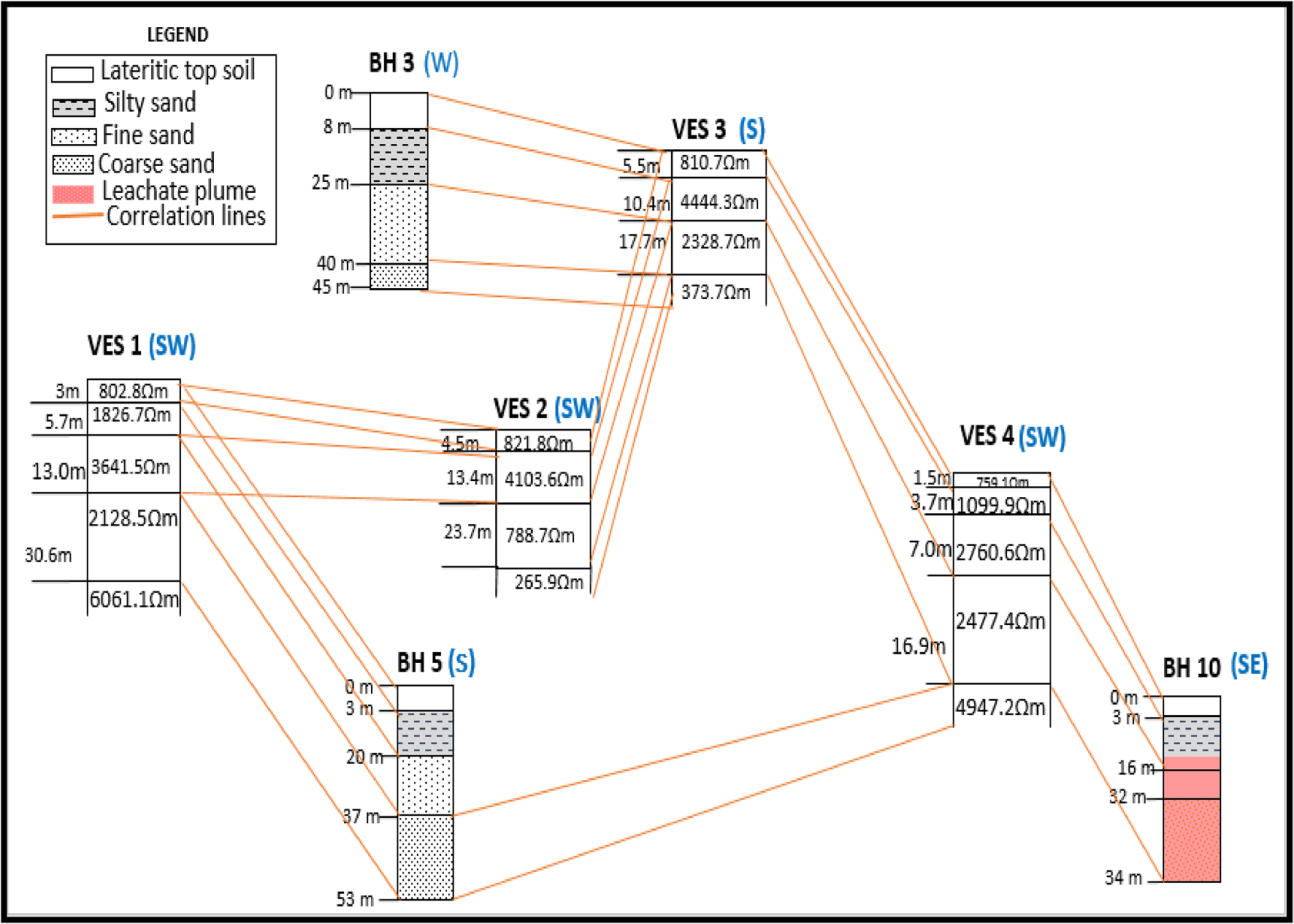
Schematic correlation of the geoelectric layers from the 4 VES and borehole logs across the study area, where the pink colour indicates borehole affected by the leachate plume.

A correlation of the vertical electrical sounding (VES) and boreholes across the study area Fig. 10 demonstrates an inferred correlation between the VES points with their apparent resistivity values and the borehole data. The VES data shows the apparent resistivity values across the area vertically, from top to the last layer, giving the spatial distribution horizontally. The borehole logs are sketches of the lithology encountered at the indicated depths on each borehole sampled. These were not drawn to scale. The geoelectric layers are interpreted sections from the Schlumberger VES data. The four geoelectric layers were correlated with the boreholes to determine the spatial distribution and variation in the apparent resistivity with depth and space, and establish their proximity to the dumpsite. This helped to establish any possible influence or relationship with the leachate from the dumpsite based on the variation of the electrical resistivity along the subsurface. Depths with the corresponding apparent resistivity value were linked. In VES 4 and borehole 5 it was observed that the apparent resistivity value decreased at depth range from 7 to 16.9 m. This is attributable to the infiltration of the leachate and the borehole being very close to the dumpsite at a distance of 0.25 km. At every depth there is a particular apparent resistivity value based on the resistivity of the area or level of saturation or, depending on the proximity to the dumpsite, the level of influence of the leachate. In addition, the value of apparent resistivity is dependent upon the amount of leachate in solution. A schematic of the correlation of the geoelectric layers from the 4 VES and borehole logs across the study area is shown in Fig. 9. The actual spatial geolocation of the boreholes (BHs) recorded using the GPS are shown in Table 1. The geospatial location of every sampling point for both the borehole and VES is shown in Fig. 4.

**Table 1:** GPS data for the geolocation of the boreholes sampled used for the correlation with the VES data.

BH	BH1	BH2	BH3	BH4	BH5	BH6	BH7	BH8	BH9
Long	6° 47′ 52″ E	6° 47′ 35″ E	6° 47′ 21″ E	6° 46′ 50″ E	6° 48′ 10″ E	6° 46′ 35″	6° 48′ 0″ E	6° 49′ 29″ E	6° 49′ 15″ E
Lat	6° 6′ 12″ N	6° 6′ 22″ N	6° 6′ 17″ N	6° 6′ 56″ N	6° 6′ 05″ N	6° 7′ 37″ N	6° 6′ 30″ N	6° 7′ 59″ N	6° 6′ 10″ N

The spatial distribution of the borehole and VES points are shown in Fig. 2. The borehole log is derived from lithology of the shallow borehole from the area.

**Figure 11:**
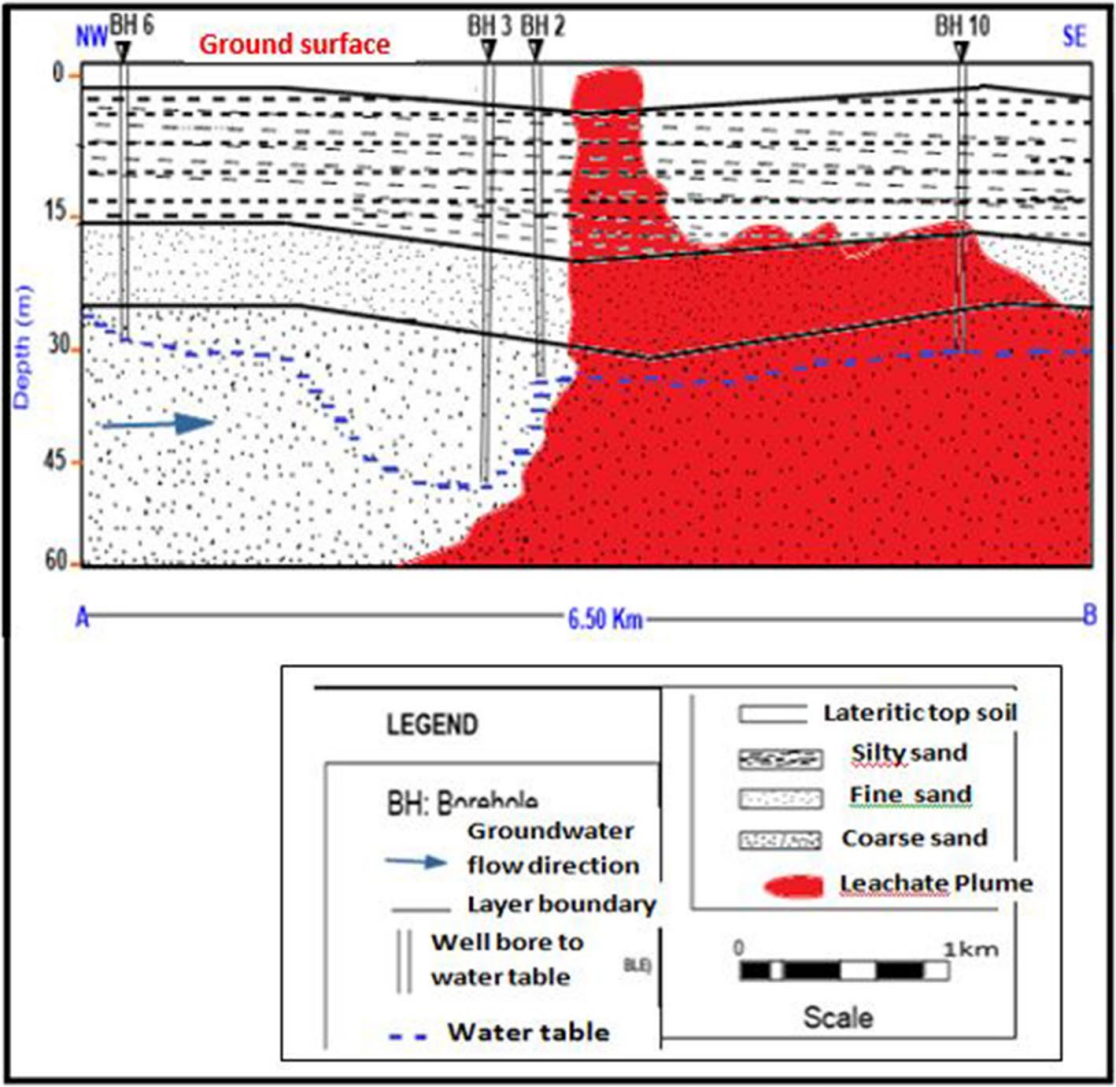
Generalized subsurface architecture of study area showing a cross section of the different measured parameters (leachate plume, water table and geoelectric layers) of the study area.

Table 2 shows the interpretation of one of the boreholes showing the lithology and the respective depth. The rest of the boreholes were interpreted and presented lithologically as shown in Fig. 11.

**Table 2:** Lithologic description of the borehole data obtained from the study area.

Soil and rock type	Depth (m)
Topsoil, reddish brown lateritic sand	0–3
Fine sand partly silt, brownish sandy clay	3–20
Fine sand, creamy white in colour	20–42
Coarse sand, white in colour	42–55

### Data integration: 2D model of sub-surface

A generalized schematic of the subsurface architecture of the study in respect of the degree of movement of the leachate away from the dumpsite is illustrated in Fig. 11. The data for the cross section was derived using the different measured parameters (leachate plume, water table and geoelectric layers) from the study area. The red colour indicates the area affected by the leachate oozing from the dumpsite. While the uncolourd part indicates the areas unaffected by the leachate. From the schematics, the leachate is shallowest at the middle. This coincides with the location closest to the dumpsite Boreholes 2, 3 and 6, located at the western half of the figure, have not been affected by the leachate. However borehole 10, located at the Eastern half of the section, has been affected by the advancing leachate plume. This is so due to two factors. One is the proximity of borehole 10 to the dumpsite, relative to the others. Secondly, the direction of groundwater flow indicates that the flow direction of the leachate is in the southeast direction which favours the eastward movement of the leachate. This conforms to the established fact that much of the leachate would tend toward the flow direction of the groundwater serving as a good solvent. This conclusion is also favoured by the local geology of the area underling the dumpsite which is characterized by a top lateritic cap underlain by fine to medium grained sandstone, both of which are porous and permeable. The geological framework of the area hence indicates the penetration of the leachate plume at varying depths from shallowest close to the dumpsite and deepening and spreading southeastwards. The groundwater from areas affected by the leachate thus become unfit for use as portable water.

## Conclusion

Geoelectrical imaging has shown good application in mapping the distribution of resistivity of the subsurface based on the content or otherwise of leachate from the dumpsite under study. Areas affected by the leachate could be inferred from the 2D inversion sections as well as the VES data: results suggest leachate migration into the subsurface as well as the surrounding soils towards the southeast direction in the study area. This is in line with the result of the vertical electrical sounding made at the dumpsite and the resistivity data. The 2D Inversion delineated contamination plumes as low resistivity zones with resistivity values ranging between 6.99 and 62.6 Ωm, from surface to variable meters depths of 0.375–3.60 m in profile 2. Profile 4 can be attributed to the leachate derived from decomposed waste of lower concentrations. On the other hand, profile 1 and profile 3 delineated contamination plumes with resistivity zones ranging between 1.24 and 36.7 Ωm, from the ground surface to varying depths.

This is possibly resulting from leachate from decomposed waste of higher concentrations. There was no evidence of possible influence on groundwater as revealed by the inversion model in profiles 2 and 4. However, the topsoil and the groundwater in profiles 1 and 3 have been affected. The geoelectric layers range from 4 to 5 and are mostly of K and H curves. The VES data for Locations 1, 2, 3, and 4 show that the depth to the water table is above 52.3 m, 41.7 m, 33.6 m, and 29.1 m, respectively.

From the generalized architectural subsurface model generated, boreholes 2, 3, and 6 have not been affected by the leachate while borehole 10 has been affected by the leachate. Consequently, given the depth of the leachate plume and the direction of flow of the conducting groundwater in the area, sinking shallow wells around the dumpsite should be avoided since the area is potentially affected by the leachate. However deeper borehole of over 100 m is advised if closer to the dumpsite. Groundwater in the area is safer if cited over 1 km away from the dumpsite.

## Data Availability

Data was acquired from field during the course of the study.

## Code availability

Industry-university support suite of software in the Department of Geology, University of Nigeria.
